# Self-Calibration of a Large-Scale Variable-Line-Spacing Grating for an Absolute Optical Encoder by Differencing Spatially Shifted Phase Maps from a Fizeau Interferometer

**DOI:** 10.3390/s22239348

**Published:** 2022-12-01

**Authors:** Xin Xiong, Chenguang Yin, Lue Quan, Ryo Sato, Hiraku Matsukuma, Yuki Shimizu, Hideaki Tamiya, Wei Gao

**Affiliations:** 1 Department of Finemechanics, Tohoku University, Sendai 980-8579, Japan; 2 The State Key Lab of Fluid Power and Mechatronic Systems, Zhejiang University, Hangzhou 310027, China; 3 Department of Human Mechanical Systems and Design, Hokkaido University, Sapporo 060-0808, Japan; 4 Magnescale Co., Ltd., Suzukawa 45, Isehara 259-1146, Japan

**Keywords:** variable-line-spacing grating, diffraction gratings, self-calibration, optical encoder, interferometry, interferometric pseudo-lateral-shearing method

## Abstract

A new method based on the interferometric pseudo-lateral-shearing method is proposed to evaluate the pitch variation of a large-scale planar variable-line-spacing (VLS) grating. In the method, wavefronts of the first-order diffracted beams from a planar VLS grating are measured by a commercial Fizeau form interferometer. By utilizing the differential wavefront of the first-order diffracted beam before and after the small lateral shift of the VLS grating, the pitch variation of the VLS grating can be evaluated. Meanwhile, additional positioning errors of the grating in the lateral shifting process could degrade the measurement accuracy of the pitch variation. To address the issue, the technique referred to as the reference plane technique is also introduced, where the least squares planes in the wavefronts of the first-order diffracted beams are employed to reduce the influences of the additional positioning errors of the VLS grating. The proposed method can also reduce the influence of the out-of-flatness of the reference flat in the Fizeau interferometer by taking the difference between the measured positive and negative diffracted wavefronts; namely, self-calibration can be accomplished. After the theoretical analysis and simulations, experiments are carried out with a large-scale VLS grating to verify the feasibility of the proposed methods. Furthermore, the evaluated VLS parameters are verified by comparing them with the readout signal of an absolute surface encoder employing the evaluated VLS grating as the scale for measurement.

## 1. Introduction

A diffraction grating is one of the key optical components in many scientific instruments such as spectrometers and optical encoders [1,2,3,4]. Diffraction gratings can be classified into two types in terms of pitch distribution; a standard grating having a constant pitch, and a variable-line-spacing (VLS) grating having a variable yet specified pitch distribution [5]. Both types can be fabricated by mechanical diamond cutting or laser interference lithography techniques [6,7,8,9]. Recently, the demand for absolute-type optical encoders is increasing, in which a VLS grating serves as a scale for position measurement [1,10,11]. With the employment of a planar VLS grating having grating pattern structures along the two orthogonal directions, in-plane absolute position measurement can be achieved [10]. However, instabilities in the mechanical manufacturing process or any distortion and imperfect wavefront control in the lithography process can result in unwanted pitch deviation of the planar VLS grating [12,13], which can influence its performance as the scale for two-dimensional absolute position measurement. Since the measurement range of the absolute optical encoder with the planar VLS grating is mainly determined by the scale size [14], a large-scale planar VLS grating is often employed. It is thus necessary to evaluate the *X*- and *Y*-directional pitch distributions of a planar VLS grating.

Several ingenious techniques and methods have been developed for the calibration of the pitches of standard gratings, and some of them can also be applied to the evaluation of a planar VLS grating. Critical-dimension atomic force microscopes or scanning electron microscopes can be used to assess the pitch variation of a grating [15,16,17,18]. However, it is not practical to measure VLS gratings in the 100 mm class due to the limited measurement throughputs and measurement ranges of these instruments. Although vacuum interferometric comparators having a high measurement throughput and a wide measurement range can be employed for the purpose [19,20], a high degree of environmental control is required. In addition, it costs too much to construct the comparators for the measurement of the two-dimensional pitch variation of a planar VLS grating. Other methods based on a long trace profiler or laser autocollimation can also be employed for the calibration of one-axis VLS grating [21,22,23]. Meanwhile, it is not an easy task to extend them for fast measurement of the two-dimensional pitch variation.

On the other hand, interferometric measurement is a possible way to solve the above-mentioned issues. The authors’ group has proposed a self-calibration method, in which the pitch deviation of a standard grating can be evaluated while self-calibrating the out-of-flatness of the reference flat in a Fizeau interferometer. In the method, the pitch deviation of the grating can be readily evaluated by using the wavefronts of the first-order diffracted beams from a grating [24,25]. The self-calibration method has been further extended to evaluate the pitch distribution of a VLS grating by linking the VLS parameters with the differential wavefronts of the first-order diffracted beams through a simple polynomial fitting process [26]. However, the coordinate in the polynomial fitting to the differential wavefronts cannot be determined accurately. As a result, the measurable area of the conventional self-calibration method has been restricted by the aperture size of the interferometer, which is usually on the order of 100 mm. Although this issue can be addressed by conducting an experiment with a large Fizeau form interferometer capable of capturing the diffracted wavefront from the whole area of a large-scale VLS grating [27], it costs too much to prepare such an interferometer in ordinary research laboratories and machine shops. Another solution for this issue is to perform stitching measurements [28,29,30]. The authors’ group has carried out stitching calibration of a long-range linear scale [31], and a linear scale in size of 105 mm over the aperture of a Fizeau interferometer (100 mm) has successfully been evaluated. However, it has been found that the influences of the grating misalignments in the Littrow configurations for capturing the wavefronts of the first-order diffracted beams cannot be easily removed. The tilt/tip/piston errors in the stitching measurement have also been found to influence the stitching accuracy [32,33].

Meanwhile, introducing an interferometric pseudo-lateral-shearing method for absolute surface metrology, in which the arithmetic operation is performed with the wavefronts (phase maps) obtained before and after the lateral shift of the surface being measured [34,35], is a possible way to retrieve all the VLS parameters of a large-scale planar VLS grating; since the coordinate in the polynomial fitting to the differential wavefronts does not need to be determined accurately in the proposed method, VLS parameters of the whole large-scale planar VLS grating can be evaluated by measuring wavefronts at different positions on the grating. In this paper, a new method inspired by the conventional pseudo-lateral-shearing method is proposed to evaluate the pitch variations of a large-scale planar VLS grating. It should be noted that the shifting error of a grating in the measurement process could affect the measurement accuracy of the pitch variation. To address the issue, the technique referred to as the reference plane technique, in which the least squares planes of the wavefronts of the first-order diffracted beams are employed to cancel the influence of the shifting error, is also introduced in this paper. At first, theoretical equations based on the conventional pseudo-lateral-shearing method are derived. After that, some numerical calculations by the derived equations are conducted to verify the feasibility of the proposed method. To further validate the performance of the newly proposed method, the pitch variation of a 150 mm size VLS grating designed for the absolute surface encoder [36] is evaluated by using a commercial Fizeau interferometer. To validate the acquired VLS parameters, experiments are also conducted by comparing them with the readouts of the absolute surface encoder employing the planar VLS grating.

## 2. Principle

### 2.1. Wavefront Measurement and VLS Parameters

**Figure 1:**
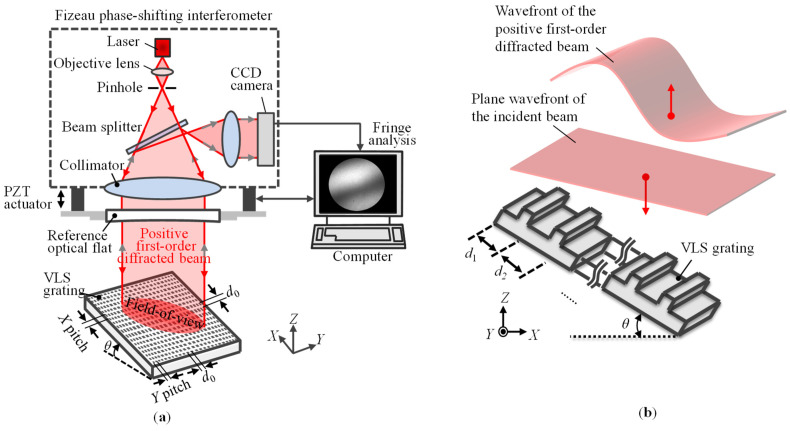
Measurement of the wavefront of the diffracted beam from a large-scale VLS grating: (**a**) Schematic of the measurement setup; (**b**) Wavefront of the diffracted beam from a typical VLS grating (The pitch variation subjects to second-order polynomial law).

The proposed method begins with the wavefront measurement of the positive and negative first-order diffracted beams from a VLS grating in Littrow configurations, in each of which the first-order diffracted beam is directly back-reflected into the direction of the incident beam. Through the phase-shifting-interferometry technique, the phase in the acquired wavefront is recorded and is further reconstructed by the phase unwrapping technique embedded in most of the commercial phase-shifting interferometers. Figure 1a shows a schematic of the wavefront measurement of a VLS grating in Littrow configuration. According to the calibration sheet provided by the manufacturer of the Fizeau interferometer, the reference flat has a small out-of-plane error that is almost like a curved surface. The *X*-directional positive and negative first-order phase outputs *I_X_*_+1_(*x*) and *I_X_*_−1_(*x*), respectively, can be expressed by the following equations:

(1) $$I_{X+1}(x)=\phi_{X+1}(x)+\phi_{Z}(x)-\phi_{R}(x)=\phi_{X+1}(x)+2\pi \frac{2e_{Z}(x)}{\lambda }\cdot \cos\theta -\frac{4\pi }{\lambda }\cdot e_{R}(x)$$

(2) $$I_{X-1}(x)=\phi_{X-1}(x)+\phi_{Z}(x)-\phi_{R}(x)=\phi_{X-1}(x)+2\pi \frac{2e_{Z}(x)}{\lambda }\cdot \cos\theta -\frac{4\pi }{\lambda }\cdot e_{R}(x)$$

where *λ* is the wavelength of the laser beam, and *θ* is the Littrow angle. *ϕ_X_*_+1_(*x*) and *ϕ_X_*_−1_(*x*) are the phase distortions in the wavefronts of the positive and negative first-order diffracted beams, respectively, caused by the pitch variation of the VLS grating. *ϕ_Z_*(*x*) and *ϕ_R_*(*x*) are the phase errors induced by the out-of-flatness errors *e_Z_*(*x*) and *e_R_*(*x*) of the VLS grating and the reference flat in the Fizeau interferometer, respectively. Figure 1b shows an example of the wavefront of the positive first-order diffracted beam from a VLS grating, whose pitch variation is subject to the second-order polynomial law. As can be seen in the figure, the wavefront of the diffracted beam will directly be affected by the pitch variation of the VLS grating. By combing Equations (1) and (2), the phase error components *ϕ_Z_*(*x*) and *ϕ_R_*(*x*) can be removed, and the following equation can be obtained:

(3) $$\phi_{X}(x)=I_{X+1}(x)-I_{X-1}(x)=\frac{4\pi }{d_{0}}\int \frac{d_{0}-d(x)}{d(x)}dx$$

where *ϕ_X_*(*x*) is the differential wavefront of the positive and negative first-order diffracted beams, and *d*(*x*) is the grating pitch at the *X*-directional absolute position along the grating. The parameter *d*_0_ is the grating pitch at *x* = 0. On the assumption that the pitch of a VLS grating changes gradually from the center (*x* = 0) to both directions along the grating symmetrically, *d*(*x*) can be described as follows by a polynomial function:

(4) $$d(x)=d_{0}+a_{1}x+a_{2}x^{2}+\ldots +a_{n}x^{n}$$

where *a_i_* (*i* = 1, 2, ···, *n*) are the coefficients. Taking into consideration that the groove line density *b*(*x*) is defined as *b*(*x*) ≈ 1/*d*(*x*) for the VLS gratings with small pitch variations, Equation (3) can be rewritten as follows:

(5) $$\phi_{X}(x)=\frac{4\pi }{d_{0}}\int \frac{d_{0}-d(x)}{d(x)}dx=4\pi \int \left(b(x)-b_{0}\right)dx≅4\pi \int \sum_{i=1}^{n}b_{i}x^{i}dx$$

where the groove line density *b*(*x*) can be expressed as follows by a polynomial function, in the same manner as *d*(*x*):

(6) $$b(x)=b_{0}+b_{1}x+b_{2}x^{2}+\ldots +b_{n}x^{n}$$

The parameters *b_i_* (*i* = 1, 2, ···, *n*) are the coefficients referred to as the VLS parameters. It should be noted that here only the *X*-directional VLS parameters are analyzed for the sake of simplicity. The calibration of the *Y*-directional VLS parameters can also be performed in the same manner as the *X*-directional VLS parameters by collecting the wavefronts of the *Y*-directional first-order diffracted beams.

### 2.2. Self-Calibration of the VLS Parameters of the Large-Scale VLS Grating

Equation (5) indicates that it is possible to retrieve the VLS parameters through polynomial fitting to the differential wavefront *ϕ_X_*(*x*) that can be obtained by the wavefront measurements of the diffracted beams. It also realizes the self-calibration of measurement, since the out-of-flatness error of the reference flat in the Fizeau interferometer can be removed by the differential operation. However, the absolute *X*-directional coordinate in the polynomial fitting in Equation (5) cannot be determined accurately since only a part of the large-scale VLS grating can be covered due to the limited aperture size of the interferometer. To explain this, now we consider the case where an offset ∆*x* exists between the actual position (*x* + ∆*x*) and the ideal position (*x*). In this case, Equation (5) becomes as follows:

(7) $$\phi_{X}(x)=4\pi \cdot \sum_{i=1}^{n}\frac{1}{i+1}b_{i}{\left(x+\Delta x\right)}^{i+1}+c$$

where *c* is the constant term after the integral operation. Performing polynomial fitting to the binomial expansion of Equation (7) only gives *b_n_* at the highest order, while the estimation of the remaining VLS parameters *b_i_* (*i* = 1, 2, ···, *n* − 1) is affected by the existence of ∆*x* (Detailed expansion of Equation (7) is presented in Appendix A). Therefore, additional wavefront information is needed to determine the remaining VLS parameters *b_i_* (*i* = 1, 2, ···, *n* − 1).

**Figure 2:**
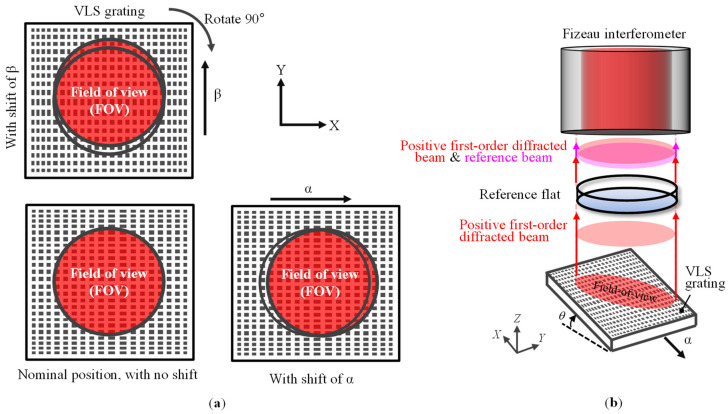
A new method integrating an interferometric pseudo-lateral-shearing method: (**a**) A schematic of the measurement procedure for calibration of the VLS parameters of the large-scale VLS grating; (**b**) A schematic showing the concept of the proposed method based on pseudo-lateral-shearing (In each of the three measurements, the interferometer is fixed with respect to the VLS grating).

To address the issue, a new method integrating an interferometric pseudo-lateral-shearing method [34] into the conventional self-calibration method for the evaluation of a planar VLS grating is proposed. The concept of the proposed method is described in Figure 2a; the VLS grating is shifted in the orthogonal directions by a distance comparable to the lateral resolution of the Fizeau interferometer. Since only the grating is shifted while the interferometer is held stationary in the setup as shown in Figure 2b, the phase error induced by the out-of-flatness error of the reference flat in the Fizeau interferometer can be canceled out when taking the difference between the measured phase outputs before and after the shift.

The differential wavefront *ϕ_X_*__shifted_(*x*), which is the difference between the first-order phase outputs *I_X_*_±1_shifted_(*x*) after the lateral shift of the grating by a distance *α* along the *X*-direction, can be described as follows:

(8) $$\phi_{X\_\mathrm{shifted}}(x)=I_{X+1\_\mathrm{shifted}}(x)-I_{X-1\_\mathrm{shifted}}(x)≅4\pi \int \sum_{i=1}^{n}b_{i,X}{\left(x+\alpha \right)}^{i}dx$$

where *b_i_*_,*X*_ (*i* = 1, 2, ···, *n*) correspond to the *X*-directional VLS parameters of the VLS grating. Consequently, by taking the difference between the differential phase outputs before and after the lateral shift, the following equation can be obtained:

(9) $$f_{X}(x)=\phi_{X\_\mathrm{shifted}}(x)-\phi_{X}(x)=4\pi \int \left(\sum_{i=1}^{n}b_{i,X}{\left(x+\alpha \right)}^{i}-\sum_{i=1}^{n}b_{i,X}x^{i}\right)dx=4\pi \int \sum_{i=1}^{n}{\bar{b}}_{i,X}x^{i-1}dx$$

where ${\bar{b}}_{i,X}$ (*i* = 1, 2, ···, *n*) is the polynomial coefficient after the binomial expansion.

**Figure 3:**
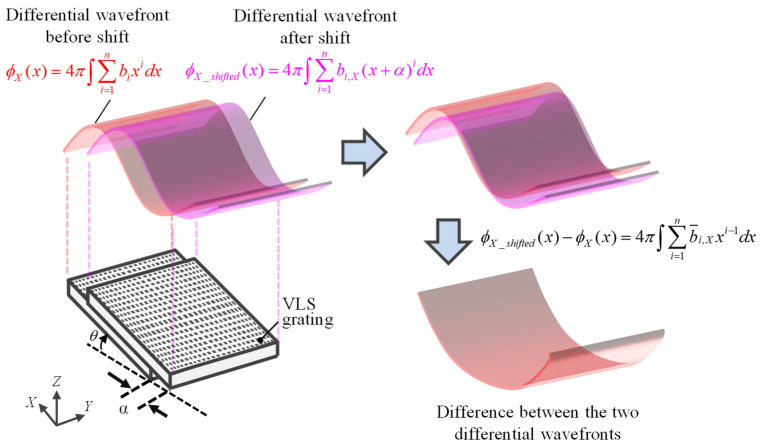
Self-calibration of the VLS parameters of the large-scale VLS grating by using the differential diffracted wavefronts before and after the lateral shift (The pitch variation of the grating is subjected to the second-order polynomial in the example).

Equation (9) leads to:

(10) $${\tilde{f}}_{X}(x)=f_{X}(x)/4\pi =\sum_{i=1}^{n}\frac{{\bar{b}}_{i,X}}{i}x^{i}+c_{1}=\sum_{i=1}^{n}{\tilde{b}}_{i,X}x^{i}+c_{1}$$

where ${\tilde{b}}_{i,X}$ (*i* = 1, 2, ···, *n*) are the corresponding polynomial coefficients after the integration. The parameter *c*_1_ is a constant term. Equation (10) indicates that all the VLS parameters can be obtained by performing polynomial fitting to the difference between the differential phase outputs before and after the lateral shift. Figure 3 shows a simple example of how additional information on VLS parameters can be obtained without prior knowledge of the coordinate system from the difference in differential phase outputs before and after the shift.

In a general case, to retrieve the VLS parameters from Equation (10), the following linear equations expressed by matrix multiplication should be solved:

(11) $$\left(\begin{array}{ccccc} \alpha & \alpha^{2} & \alpha^{3} & \cdots & \alpha^{n}\\ 0 & \frac{1}{2}C_{2}^{1}\alpha & \frac{1}{2}C_{3}^{1}\alpha^{2} & \cdots & \frac{1}{2}C_{n}^{1}\alpha^{n-1}\\ 0 & 0 & \frac{1}{3}C_{3}^{2}\alpha & \cdots & \frac{1}{3}C_{n}^{2}\alpha^{n-2}\\ \vdots & \vdots & \vdots & \ddots & \vdots \\ 0 & 0 & 0 & \cdots & \frac{1}{n}C_{n}^{n-1}\alpha \end{array}\right)\cdot \left(\begin{array}{c} b_{1,X}\\ b_{2,X}\\ b_{3,X}\\ \vdots \\ b_{n,X} \end{array}\right)=\left(\begin{array}{c} {\tilde{b}}_{1,X}\\ {\tilde{b}}_{2,X}\\ {\tilde{b}}_{3,X}\\ \vdots \\ {\tilde{b}}_{n,X} \end{array}\right)$$

According to Equation (11), the solution is overdetermined, since the VLS parameter *b_n_*_,*X*_ can be readily obtained by using the difference between the differential phase outputs before the lateral shift. The corresponding system of simultaneous linear equations could be expressed as:

(12) AB=S

where ***A*** is an *n*-by-*n*-coefficient array, ***B*** is an *n*-element vector containing the VLS parameters of the grating, and ***S*** is an *n*-element vector containing the coefficients of the polynomial fitting to the differential wavefront from Equation (10). The solution to Equation (12) that minimizes the error using the least-square technique is well known to be expressed as follows:

(13) $$B=A^{-1}S$$

Consequently, all the VLS parameters can be evaluated according to the coefficients in vector ***B***. It should be noted that the *Y*-directional analysis can also be performed by collecting the *Y*-directional diffracted beams, in the same manner as the *X*-directional analysis.

**Figure 4:**
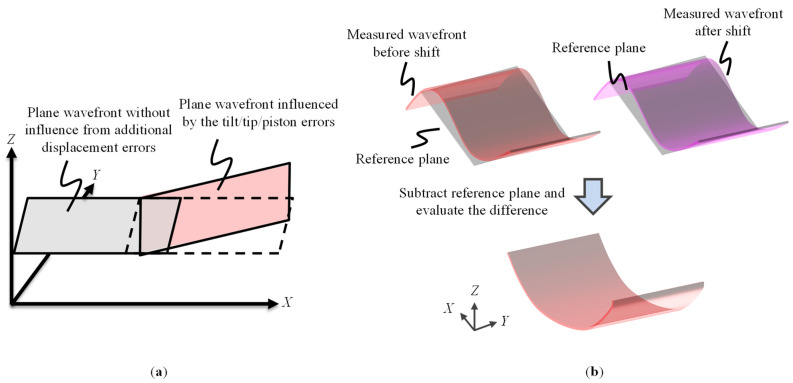
The proposed reference plane technique: (**a**) An example showing the plane wavefront influenced by the tilt/tip/piston error after the shift; (**b**) A schematic showing the procedure of the proposed reference method.

It should be noted that Equation (8) is valid under the ideal condition without any misalignments of the grating with respect to the Fizeau interferometer. In a practical case, additional positioning errors (tilt/tip/piston errors) could be generated during the shifting process of the VLS grating. Figure 4a shows a schematic of the least squares planes of the wavefronts influenced by the tilt/tip/piston errors. To address the issue, a new technique referred to as the reference plane technique is employed. In the technique, the least square planes (reference planes) of the measured wavefronts before and after the shift are utilized. Figure 4b shows the procedure of the proposed reference plane technique. It should be noted that the operation of subtracting the reference plane can be readily performed by the software embedded in a commercial Fizeau interferometer. The detail of the proposed reference method is included in Appendix B for the sake of simplicity.

## 3. Simulations

Computer simulations are performed to verify the feasibility of the proposed method. In the simulations, the wavelength of the laser source of the phase-shifting interferometer is set to be 632.8 nm. For the sake of simplicity, one-dimensional scale grating whose grating pitch varies only in the *X*-direction is considered. It should be pointed out that in a two-dimensional simulation the fitting error tends to increase as more parameters are involved. However, this point will not be discussed here in order not to lose the focus of this paper. Regarding the experimental result in the previous research by the authors’ group [26], the out-of-flatness errors of the grating substrate and the reference flat are expressed by the following polynomial equations:

$$e_{Z}(x)=19.03-86.23x-179.43x^{2}+132.15x^{3}+147.87x^{4}\;[\mathrm{nm}]$$

$$e_{R}(x)=25.13+29.73x^{2}\;[\mathrm{nm}]$$

**Figure 5:**
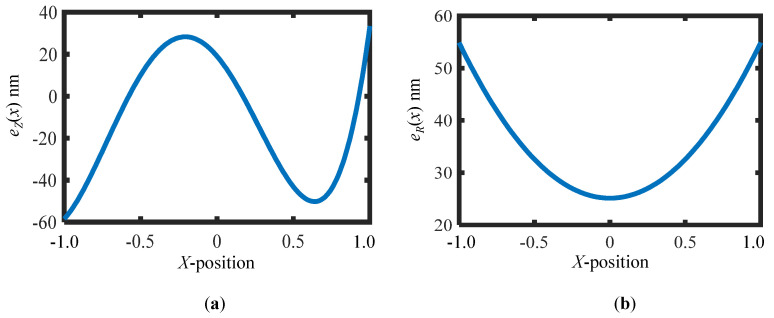
Simulated form of the VLS grating and the reference flat: (**a**) Simulated out-of-flatness of the VLS grating; (**b**) Simulated out-of-flatness of the reference flat.

Figure 5a,b show the out-of-flatness of the VLS grating and the reference flat, respectively, assumed in the simulation. It should be noted that the *X*-coordinate *x* is normalized to be [−1, 1] from the range of [−60, 60], which corresponds to the simulation range of the VLS grating, for the sake of simplicity.

**Figure 6:**
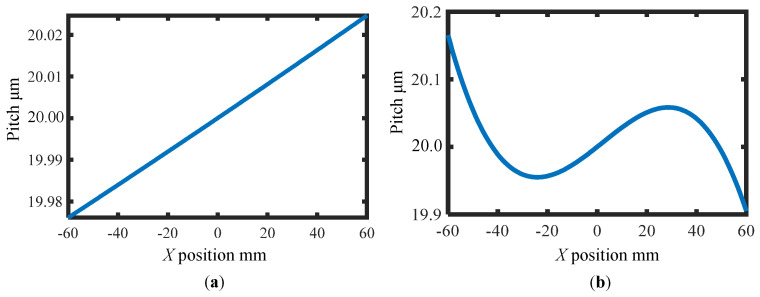
Simulated pitch variation of the VLS grating in the two cases. (**a**) Simulated pitch variation of the VLS grating in case one; (**b**) Simulated pitch variation of the VLS grating in case two.

**Figure 7:**
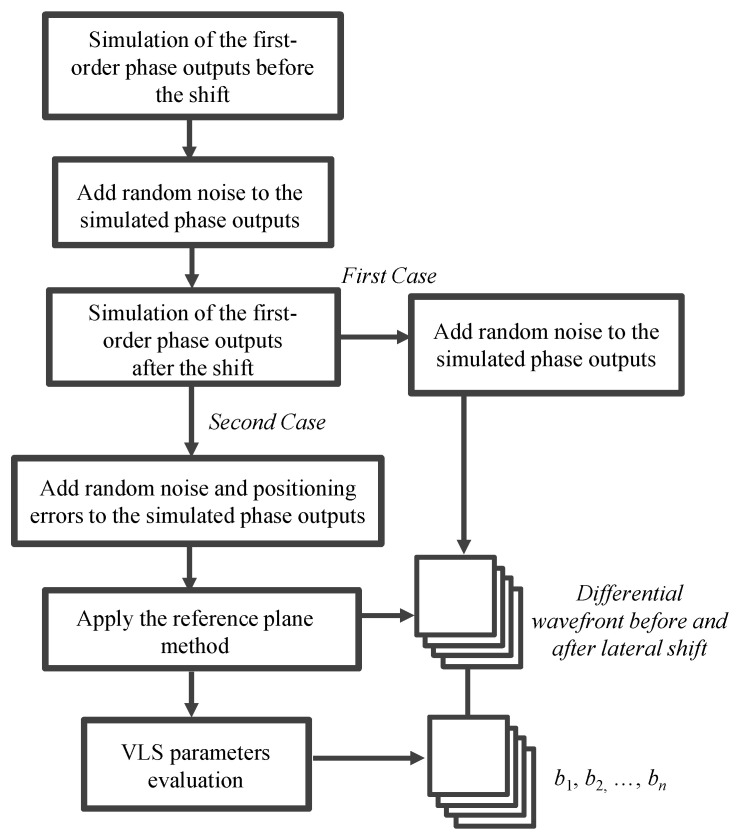
Flow diagram of the simulation procedure.

The procedure of the simulation is as follows: In the first step, the phase distortions *ϕ_X±_*_1_(*x*) caused by the pitch variation of the VLS grating before the grating shift are calculated by considering the pitch variation. Then, the first-order phase outputs *I_X_*_±1_(*x*) are simulated by the given out-of-flatness errors of the grating *e_Z_*(*x*) and the interferometer reference flat *e_R_*(*x*), as well as the simulated phase distortions *ϕ_X±_*_1_(*x*). In the second step, random noise is added to the simulated phase outputs. Noise components having Gaussian distribution with mean *I_i_*(*x*) and standard deviation *γ*|*I_i_*(*x*)| (*i* = *x* ± 1) are added to the phase outputs. To avoid underestimating the influence of the noise components, *γ* was set to 5%. In the third step, phase distortions *ϕ_X±_*_1_(*x*) after the shift are simulated, and then the first-order phase outputs after the grating shift are simulated in the same manner as the first step. To verify the feasibility of the proposed reference plane technique, random positioning errors are added to the simulated phase outputs after the grating shift. Meanwhile, random noises with Gaussian distribution are also added to the phase outputs after the grating shift. As the final step, we use the proposed method to retrieve the VLS parameters to verify the feasibility of the model. In the following, two cases having different pitch variations shown in Figure 6 are treated in the simulation. In addition, cases with and without positioning errors are considered. The procedure of the simulation process is summarized in Figure 7.

(a) First case: *b*(*x*) in the second-order polynomials

*b*(*x*) = *b*_0_ + *b*_1_*x*+ *b*_2_*x*^2^, while *b*_0_, *b*_1_ and *b*_2_ were set to be 50 lines/mm, −1.01 × 10^−3^ lines/mm^2^ and −2.38 × 10^−7^ lines/mm^3^, respectively.

**Figure 8:**
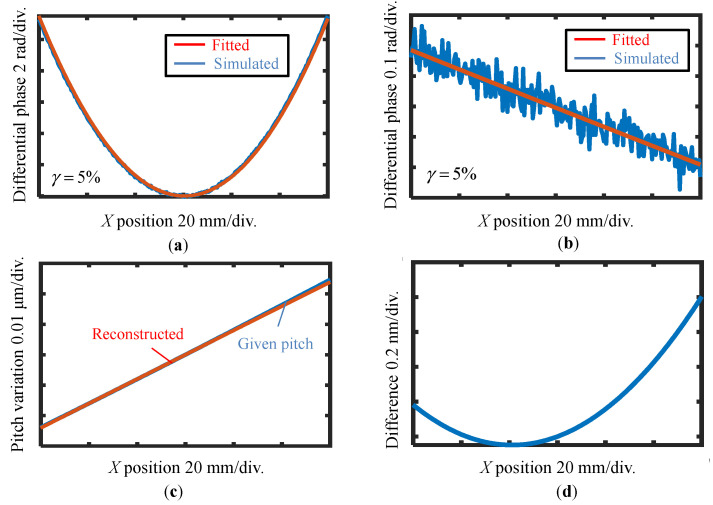
Reconstructed pitch distribution result of case one using the proposed method without applying the reference plane technique. (**a**) Evaluated differential phase outputs and its fitting result before the shift with 5% noise level; (**b**) Evaluated differential phase outputs and its fitting result after the shift with 5% noise level; (**c**) Given pitch in simulation and reconstructed pitch variation of VLS grating; (**d**) Difference between the simulation and the reconstruction result.

At first, the case in Figure 6a is simulated without applying the reference plane technique. Figure 8a shows the simulated differential phase output and its fitting result before the shift with a 5% noise level. The best third-order polynomial fitting result of the differential wavefront before the shift is shown in Figure 8a. Then, the best second-order polynomial fitting is applied to the differential wavefront before and after the shift, as shown in Figure 8b. At last, the VLS parameters are calculated according to the fitting results, and the reconstructed pitch variation is shown in Figure 8c, while the residual between the given pitch and the reconstructed one is shown in Figure 8d. From the analysis result, the retrieved parameters are found to be close to the original values even under the influence of noise components, which results in reconstruction errors of ∆*b*_1_ = 1.01 × 10^−5^ lines/mm^2^ (1%) and ∆*b*_2_ = 1.67 × 10^−7^ lines/mm^3^ (70%), respectively. From the results, it could be concluded that the estimation error of *b*_1_ is approximately 1% (∆*b*_1_/*b*_1_) while it is comparable to parameter *b*_2_. The amount of the error is mainly caused by the added random noise; in the actual measurement, the noise level is believed to be better than the 5% level. Through the simulation, it is verified that the proposed method can retrieve the relatively small VLS parameters.

(b) Second case: *b*(*x*) in the third-order polynomials

*b*(*x*) = *b*_0_ + *b*_1_*x* + *b*_2_*x*^2^ + *b*_3_*x*^3^, while *b*_0_, *b*_1_, *b*_2_ and *b*_3_ were set to be 50 lines/mm, −7.30 × 10^−3^ lines/mm^2^, −2.38 × 10^−5^ lines/mm^3^, and 3.53 × 10^−6^ lines/mm^4^, respectively.

**Figure 9:**
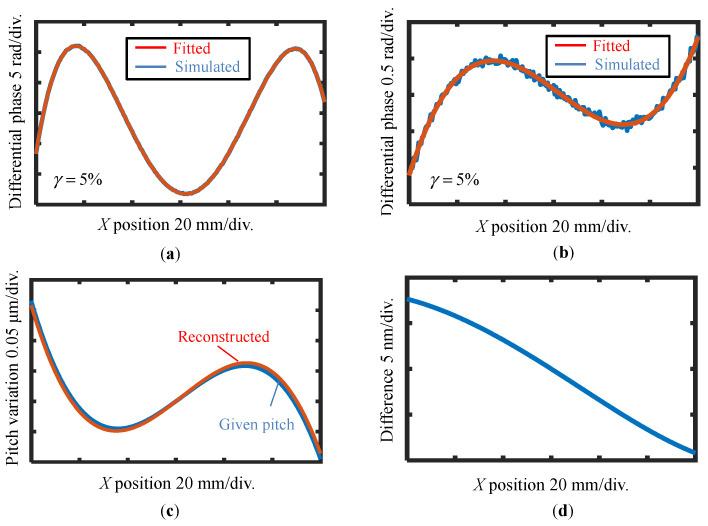
Reconstructed pitch distribution result of case two by using the proposed pseudo-lateral-shearing and reference plane technique: (**a**) Evaluated differential phase outputs and its fitting result before the shift with 5% noise level; (**b**) Evaluated differential phase outputs and its fitting result after the shift with 5% noise level; (**c**) Given pitch in simulation and reconstructed pitch distribution of VLS grating; (**d**) Difference between the simulation and the reconstruction result.

In the second case, the case in Figure 6b is simulated. In addition, the additional errors in the lateral shift are considered, and the proposed reference plane technique is applied to reduce the influences of these errors. In the simulation process, random values are assigned to the error coefficients *a_X_*_±1_, *b_X_*_±1_, and *c_X_*_±1_, and the corresponding additional displacement errors are added to the simulated phase outputs after the lateral shift. Figure 9a,b show the polynomial fitting result of the differential wavefronts with a noise level of 5%.

Figure 9c,d show the reconstructed pitch variation and difference with the proposed method, respectively. The reconstruction errors are found to be ∆*b*_1_ = 3 × 10^−4^ lines/mm^2^, ∆*b*_2_ = 2.85 × 10^−7^ lines/mm^3^ (4%), and ∆*b*_3_ = 1.16 × 10^−8^ lines/mm^4^ (1%), respectively. From the reconstruction results, the estimation error of the VLS parameter *b*_1_ is evaluated to be approximately 4% (∆*b*_1_/*b*_1_), while those of *b*_2_ and *b*_3_ are found to be less than 1%. The feasibility of the proposed method is thus verified under the condition of additional positioning errors and noise components. With the proposed reference plane technique, the additional positioning errors generated in the shifting process can be greatly reduced without employing additional compensating instruments.

## 4. Experiments and Discussions

### 4.1. Evaluation of the Large-Scale VLS Grating

**Figure 10:**
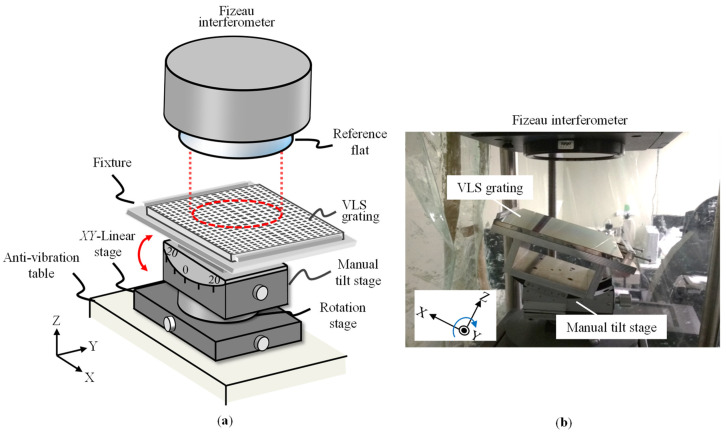
Experiment setup: (**a**) Schematic of the experimental setup with a commercial Fizeau interferometer; (**b**) Photograph of the experiment setup.

The feasibility of the proposed method is verified through experiments. Figure 10a,b show a schematic and a photograph of the experimental setup. The experiment was conducted in a room with careful temperature, humidity, and vibration control. A commercial Fizeau interferometer (Verifire^TM^, Zygo Corp., Middlefield, CT, USA) with a laser source having a wavelength of 632.8 nm was employed. The Fizeau interferometer was equipped with a reference flat in a diameter of 102 mm with an out-of-flatness of better than *λ*/20. It should be noted that the size of the reference flat directly determines the maximum measurement range of a VLS grating in a single measurement. The vertical resolution of the Fizeau interferometer was 0.05 nm. In the Fizeau interferometer, an image sensor having pixels of 480 × 640 was employed; the corresponding lateral resolution can be calculated to be 223 μm. The horizontal resolution governs the measurement interval of the wavefronts of the diffracted beams.

A VLS grating in a size of 152.1 mm × 152.1 mm, which was fabricated for an absolute-type planar encoder by using the holographic technique, was calibrated in the experiment. For the alignment of the VLS grating in Littrow configurations, a precision manual tilt stage (TS-211, Chuo Precision Industrial Co., Ltd., Tokyo, Japan) having a travel range of ±20° was employed. The grating was mounted on the tilt stage by using a grating holder, which was prepared carefully so that the rotational center of the tilt stage would rest on the grating surface. The Littrow angle of the VLS grating was determined to be 18.4°, regarding the central pitch of the VLS grating (1000 nm). The tilt stage with the VLS grating was mounted on a manual *XY*-stage to give a lateral shift to the VLS grating with respect to the Fizeau interferometer.

**Figure 11:**
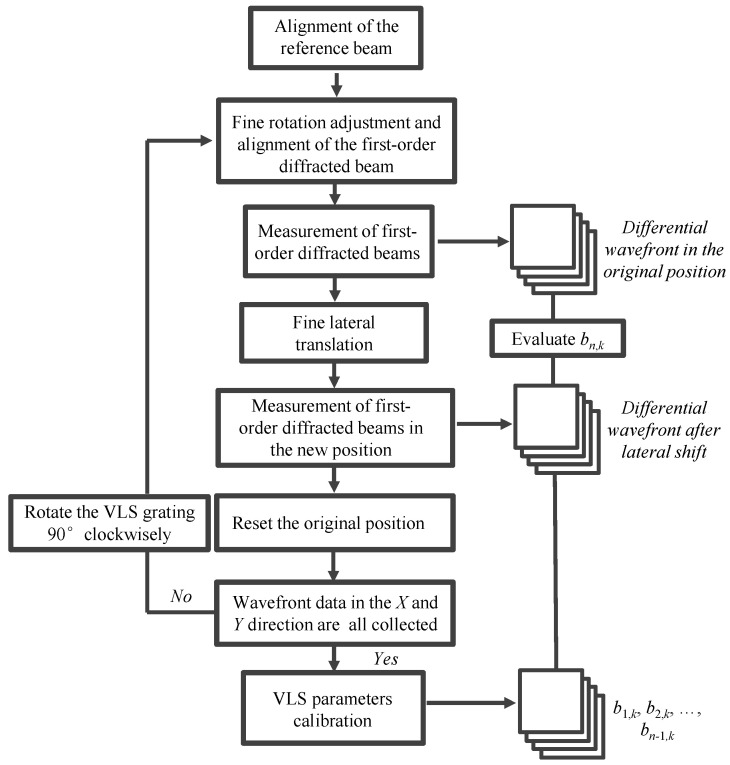
Flow diagram indicating the measurement procedure for the self-calibration of the large-scale VLS grating.

**Figure 12:**
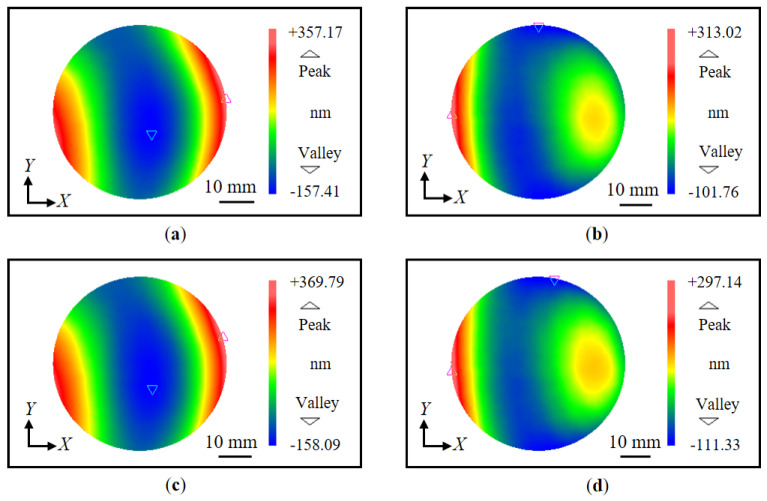
Measured diffracted wavefronts before and after shift (*X*-direction): (**a**) Measured positive first-order diffracted wavefront before the shift; (**b**) Measured negative first-order diffracted wavefront before the shift; (**c**) Measured positive first-order diffracted wavefront after the shift; (**d**) Measured negative first-order diffracted wavefront after the shift.

**Figure 13:**
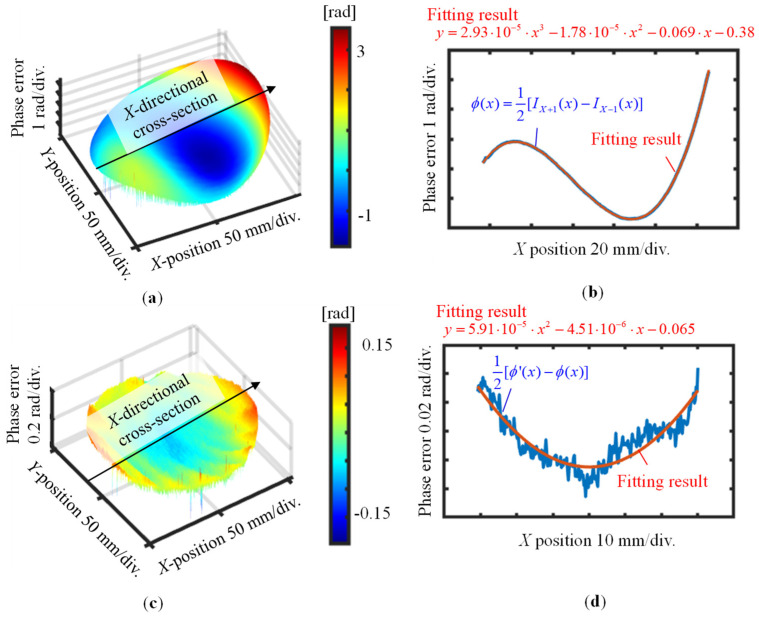
Measured first-order differential diffracted wavefronts before and after shift (*X*-direction): (**a**) Measured first-order differential diffracted wavefront before the shift; (**b**) *X*-directional cross-sectional profile of the differential wavefront in (**a**); (**c**) Difference of the first-order differential diffracted wavefront before and after the shift; (**d**) *X*-directional cross-sectional profile of the differential wavefront in (**c**).

Figure 11 shows the measurement procedure of the proposed method. Initially, the wavefronts of the *X*-directional first-order diffracted beams were measured. Attention was paid to arranging the number of fringes to be as equal as possible to decrease the influence of the out-of-flatness of the VLS grating. Five repetitive trials were conducted to gather data sets of the wavefront in each diffracted beam, and the mean of these data sets was used for further analysis to reduce the influences of environmental disturbances. Figure 12a,b show the wavefront data of the *X*-directional positive and negative first-order diffracted beams, respectively, obtained before the grating shift. The VLS grating was then slightly shifted along the *X*-direction in a step of 10 μm. Figure 12c,d show the wavefront data of the *X*-directional positive and negative first-order diffracted beams, respectively, after the shift. It should be noted that the least squares plane is removed from the wavefronts in the figures by the proposed reference plane technique. Figure 13a represents the differential wavefront of the positive and negative first-order diffracted beams before the shift, while Figure 13b shows the central cross-section of the differential wavefront.

A third-order fitting result, which is obtained on the assumption that the pitch distribution of the VLS grating is subjected to the second-order polynomial, is also plotted in the figure. Based on Equation (5), the VLS parameter *b*_2_ was estimated to be 1.39 × 10^−5^ lines/mm^3^. Figure 13c shows the differential wavefronts before and after the shift, and Figure 13d shows its central cross-section and its second-order polynomial fitting result. From the fitting results, the parameter *b*_1_ was estimated to be 2.54 × 10^−5^ lines/mm^2^ based on Equations (11)–(13). It should be noted that higher-order polynomials could also be applied to fit the differential result, depending on the forms of differential wavefronts.

**Figure 14:**
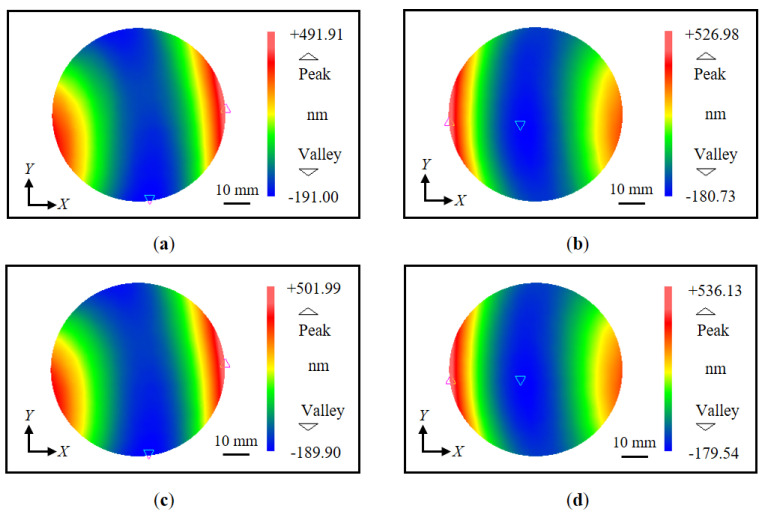
Measured diffracted wavefronts before and after shift (*Y*-direction): (**a**) Measured positive first-order diffracted wavefront before the shift; (**b**) Measured negative first-order diffracted wavefront before the shift; (**c**) Measured positive first-order diffracted wavefront after the shift; (**d**) Measured negative first-order diffracted wavefront after the shift.

**Figure 15:**
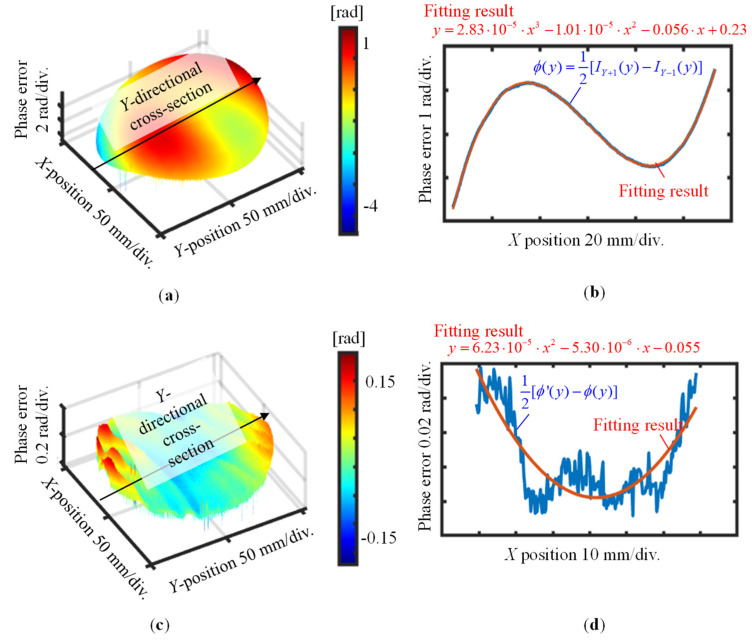
Measured first-order differential diffracted wavefronts before and after shift (*Y*-direction): (**a**) Measured first-order differential diffracted wavefront before the shift; (**b**) *Y*-directional cross-sectional profile of the differential wavefront in (a); (**c**) Difference of the first-order differential diffracted wavefront before and after the shift; (**d**) *Y*-directional cross-sectional profile of the differential wavefront in (**c**).

After turning the grating 90 degrees about the *Z*-axis in the counterclockwise direction, the *Y*-directional pitch variation of the VLS grating was also evaluated, in the same manner as the X-directional pitch variation. Figure 14 and Figure 15 provide a summary of the measurement results. For the *Y*-directional grating pitch, the VLS parameters *b*_1_ and *b*_2_ were evaluated to be 2.70 × 10^−5^ lines/mm^2^ and 1.35 × 10^−5^ lines/mm^3^, respectively. The above evaluated VLS parameters of the large-scale VLS grating with one cross-section analysis was summarized in Table 1.

**Figure 16:**
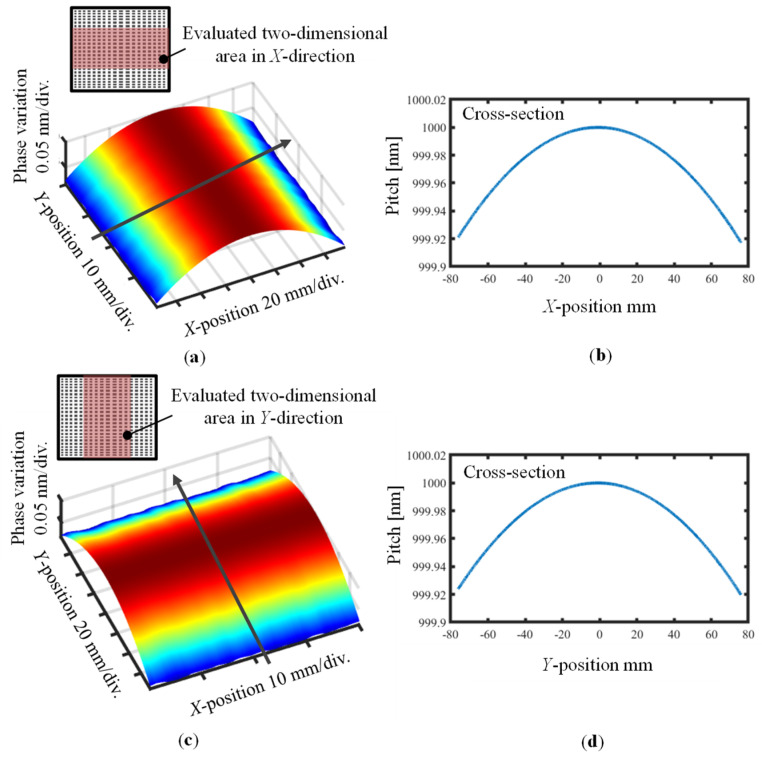
Evaluated two-dimensional pitch variations of the large-scale VLS grating: (**a**) *X*-directional two-dimensional pitch variations; (**b**) *X*-directional central cross-section pitch distribution; (**c**) *Y*-directional two-dimensional pitch variations; (**d**) *Y*-directional central cross-section pitch distribution.

Finally, the two-dimensional pitch distributions were reconstructed by using each cross-section of the obtained differential wavefront. Figure 16a,c show the two-dimensional pitch distributions while Figure 16b,d show the central cross-sections of the VLS grating along the *X*- and *Y*-directions, respectively, in the area of 40 mm × 152.1 mm reconstructed by the obtained VLS parameters. The mean values of the VLS parameters *b*_1_ and *b*_2_ are summarized in Table 2. The strong consistency between the pitch distributions in the two directions in the analyzed region and the uniform shape of the distribution map demonstrate the validity of the proposed method.

### 4.2. Comparison Results and Discussions

**Figure 17:**
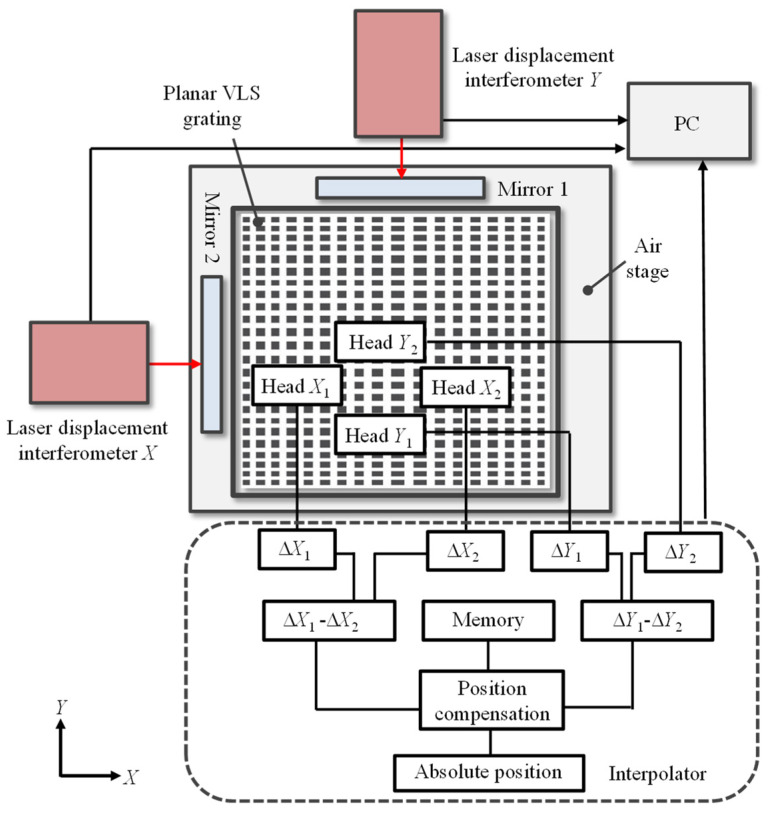
The experimental setup for the evaluation of the reading errors of the optical head in the absolute planar encoder.

A comparative experiment was also conducted by recording the reading error of an absolute optical encoder [36], in which the calibrated VLS grating was employed as the scale. Figure 17 shows a schematic of the experimental setup. The absolute optical encoder consisted of the calibrated VLS grating, two optical heads, and an interpolator for the determination of the absolute location based on the readouts from the two optical heads. In the experimental setup, the VLS grating was placed on an *XY* air-bearing stage so that the optical heads could scan the grating surface in the *X*- and *Y*-directions. A measurement range was 100 mm along both the *X*- and *Y*-directions. The separation distance of the two reading heads was arranged to be 5 mm in the experiment. Two laser interferometers were also employed as a reference for the measurement of the stage displacement. At each stage displacement in a step of 1000 nm, corresponding to the central grating pitch, the readouts of the optical head and the laser interferometer were recorded. The comparison experiment was conducted in a room with temperature and humidity control. The temperature was controlled to be 23 ± 0.05°.

**Figure 18:**
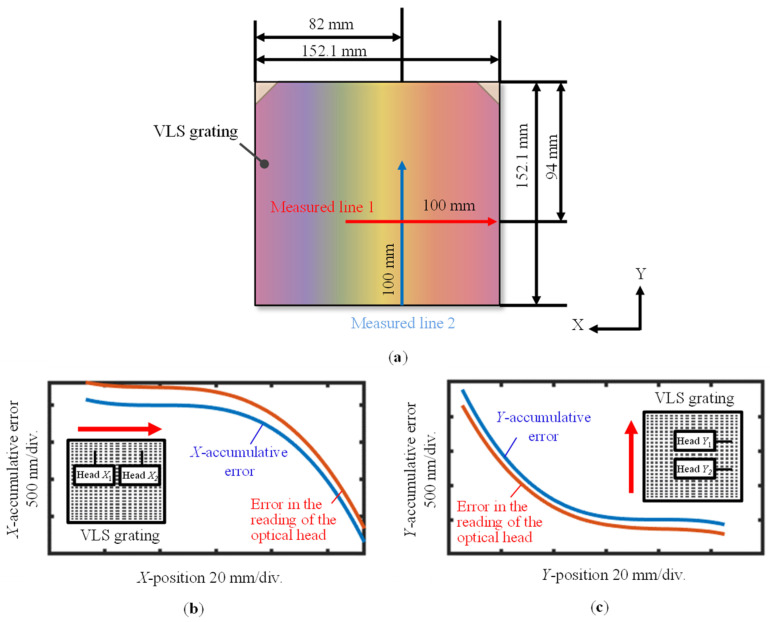
Measurement of the encoder errors of the absolute planar encoder and the comparison results: (**a**) Scanning lines along the VLS grating; (**b**) *X*-direction comparison result; (**c**) *Y*-direction comparison result.

**Figure 19:**
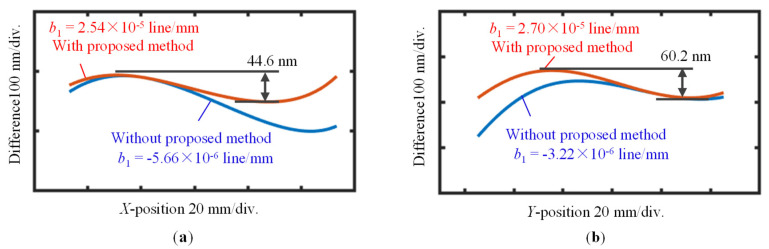
Differences between the encoder error of the absolute planar encoder and the accumulative results evaluated by using the assessed VLS parameter: (**a**) Differences between the comparison results along the *X*-direction with and without applying the proposed method; (**b**) Differences between the comparison results along the *Y*-direction with and without applying the proposed method.

Figure 18a shows the positions of the scanning lines along the VLS grating, and Figure 18b,c show the *X*- and *Y*-directional accumulative errors of the absolute encoder readouts, respectively. For the comparison, the accumulative errors calculated by using the retrieved VLS parameters are also plotted in the figures. The two comparison results were shifted along the vertical direction arbitrarily for the sake of clarity. The mean values of the VLS parameters obtained in the analyzed two-dimensional area were used for the evaluation of the accumulative errors. It should be noted that the reference plane technique was applied to obtain both *X*- and *Y*-directional VLS parameters considering the possible additional installation tilts/piston errors. Figure 19a,b show the differences between the measured accumulative errors of the encoder and the calculated accumulative errors using the evaluated VLS parameters, which were obtained with and without implementing the pseudo-lateral-shearing method, respectively.

The differences between the accumulated error calculated using the assessed VLS parameters and the results acquired by the optical encoder system were found to be less than ±50 nm over a range of 100 mm, showing that the VLS parameters were successfully estimated. Moreover, the reduced difference after using the proposed method integrating the pseudo-lateral-shearing method and the reference plane technique demonstrated a better characterization of the VLS parameter *b*_1_. The proposed method has successfully retrieved the tiny VLS parameters of the large-scale VLS grating. The peak-to-valley (PV) values of the differences between the encoder error and the accumulative errors estimated by using the assessed VLS parameters were reduced to approximately ±20 nm (*X*-direction) and ±30 nm (*Y*-direction) over a 100 mm measurement range.

Considering the precision positioning applications such as semiconductor manufacturing, the required positioning accuracy is below ±50 nm over the range of hundred millimeters displacement level and thus the calibrated results can be used for the compensation for the absolute optical encoder [36]. In addition, the influence of the removal of the reference plane on the evaluation of the VLS parameter *b*_1_ can be neglected considering the small transverse distance. The impact of the small error in the evaluated *b*_1_ on the performance of the absolute encoder will be investigated in future work.

It should be pointed out that the influence brought by different mountings of the grating in the Littrow setup, as well as the air stage, can be ignored. Since the influence of the out-of-flatness of the grating can be eliminated by the proposed self-calibration method, the interferential-scanning principle could ensure high-accuracy position measurement even with a slightly deformed grating surface. Moreover, the uncertainty caused by the polynomial fitting is relatively small, and the amount of noise can be suppressed by experimenting with well-controlled environmental conditions [37]. Employing a two-dimensional polynomial fitting could be considered to fasten the evaluation process based on the proposed approach, though the uncertainties brought by the two-dimensional polynomial fitting need to be evaluated regarding tiny VLS parameters. Meanwhile, although the VLS grating could also be calibrated by using the calibration setup, the proposed method using the Fizeau interferometer can achieve fast and high accuracy calibration over a large area of the VLS grating with modest investment and experiment setting efforts. This proposed technique thus solves a classical problem in the assessment of the pitch distributions of large-scale planar VLS gratings in an absolute way, with high sensitivity, and with modest additional experimental requirements and data processing effort. It would also be helpful for evaluating large VLS gratings with larger pitch variation, which is effective for improving the sensitivity and measurement accuracy of next-generation absolute optical encoders.

## 5. Conclusions

A new method for the self-calibration of the pitch distribution of a large-scale VLS grating for an absolute optical encoder by using a Fizeau phase-shifting interferometer has been proposed. In the method, the wavefronts of the positive and negative first-order diffracted beams before and after the slight shift of the grating under measurement have been employed to retrieve the VLS parameters, which represent the pitch variation of the VLS grating. By fitting a polynomial function to the differential wavefronts of the positive and negative first-order diffracted beams before and after the shift, the VLS parameters could have been readily evaluated in a short time. The proposed method also enables the characterization of the VLS parameters of the VLS grating when the size of the grating exceeds the aperture size of the employed interferometer. Meanwhile, the misalignment in the shifting of the grating could influence the evaluation of the VLS parameters. To address this issue, a reference plane technique has also been proposed. The feasibility of the proposed method has been demonstrated by both theoretical equations and simulations. Furthermore, to verify the feasibility of the proposed method, an experiment has been conducted by using a planar VLS grating of 150 mm size. By using the proposed method, the VLS parameters of the grating for both *X* and *Y* directions have successfully been evaluated by shifting the grating with a small distance along with the *X*- and *Y*- directions. The two-dimensional pitch distributions of the VLS grating have also been readily reconstructed in a large area.

To further confirm the validity of the proposed method, a comparison experiment has also been performed to compare the measurement error of the absolute optical encoder employing the tested grating with the pitch deviation of the VLS grating obtained by using the proposed method. Experimental results have revealed that the encoder error has mainly been caused by the pitch variation of the grating. The difference between the measured accumulative encoder error and the accumulative errors calculated by the evaluated VLS parameters is less than 50 nm in the range of 100 mm, confirming the validity of the proposed method. It should be pointed out that the paper mainly focused on the proposal of the calibration theory and primary verification. Future work includes the comprehensive uncertainty analysis of the proposed method by considering all the uncertainty sources such as the one-dimensional and two-dimensional polynomial fitting uncertainties, the setting error of the VLS grating inclination angle, and the misalignment of the wavefronts. In addition, improvement of the measurement procedure, calibration of the tilt of the VLS grating in the lateral shift by using an autocollimator and minimizing the tilt errors, as well as compensation of the encoder error by using the evaluation results will also be considered as the next steps of the research.

## Figures and Tables

**Table 1:** Evaluated VLS parameters of the large-scale VLS grating. (One cross-section analysis).

	Symbol	Values
VLS parameters (*X*-direction)	*b*_1,*X*_	2.54 × 10^−5^ lines/mm^2^
	*b*_2,*X*_	1.39 × 10^−5^ lines/mm^3^
VLS parameters (*Y*-direction)	*b*_1,*Y*_	2.70 × 10^−5^ lines/mm^2^
	*b*_2,*Y*_	1.35 × 10^−5^ lines/mm^3^

**Table 2:** Evaluated VLS parameters of the large-scale VLS grating. (Two-dimensional area analysis).

	Symbol	Mean Values	Standard Deviation
VLS parameters (*X*-direction)	*b*_1,*X*_	2.54 × 10^−5^ lines/mm^2^	1.19 × 10^−6^ lines/mm^2^
	*b*_2,*X*_	1.38 × 10^−5^ lines/mm^3^	2.59 × 10^−7^ lines/mm^3^
VLS parameters (*Y*-direction)	*b*_1,*Y*_	2.61 × 10^−5^ lines/mm^2^	9.17 × 10^−6^ lines/mm^2^
	*b*_2,*Y*_	1.33 × 10^−5^ lines/mm^3^	4.07 × 10^−7^ lines/mm^3^

## Data Availability

Not applicable.

## Appendix A. Expansion of Equation (7)

According to Equation (7), we have:

(A1) $$\begin{array}{ll} \phi_{X}(x) & =4\pi \cdot \sum_{i=1}^{n}\frac{1}{i+1}b_{i}{\left(x+\Delta x\right)}^{i+1}+c\\ & =4\pi \cdot [\frac{1}{2}b_{1}{\left(x+\Delta x\right)}^{2}+\frac{1}{3}b_{2}{\left(x+\Delta x\right)}^{3}+\ldots +\frac{1}{n}b_{n-1}{\left(x+\Delta x\right)}^{n}+\frac{1}{n+1}b_{n}{\left(x+\Delta x\right)}^{n+1}]+c \end{array}$$

Through the binomial expansion of Equation (A1), it leads to:

(A2) $$\begin{array}{ll} \phi_{X}(x)= & 4\pi \cdot [x\cdot (\sum_{i=1}^{n}\frac{1}{i+1}C_{i+1}^{1}b_{i}\Delta x^{i})+x^{2}\cdot (\sum_{i=1}^{n}\frac{1}{i+1}C_{i+1}^{2}b_{i}\Delta x^{i-1})+\ldots +x^{n}\cdot (\sum_{i=n-1}^{n}\frac{1}{i+1}C_{i+1}^{n}b_{i}\Delta x^{i+1-n})\\ & +x^{n+1}\cdot b_{n}\cdot \frac{1}{n+1}]+c \end{array}$$

Equation (A2) reveals that when fitting the *n* + 1 degree polynomial to the obtained differential wavefront, only the highest order term *b_n_* can be evaluated accurately, while other VLS parameters are coupled with the factor ∆*x* and are difficult to separate.

## Appendix B. The Reference Plane Technique

Taking the *X*-directional analysis as an example. The *X*-directional first-order phase outputs after the shift ${\tilde{I}}_{X\pm 1\_\mathrm{shifted}}(x,y)$ under the influence of the additional positioning error can be expressed by the following equation:

(A3) $${\tilde{I}}_{X\pm 1\_\mathrm{shifted}}(x,y)=I_{X\pm 1\_\mathrm{shifted}}(x,y)+a_{X\pm 1}x+b_{X\pm 1}y+c_{X\pm 1}$$

where *a_X_*_±1_, *b_X_*_±1_, and *c_X_*_±1_ are the coefficients of tilt, tip and piston errors in the corresponding measurement, respectively. The first term *I_X_*_±1_shifted_(*x*,*y*) in the equation is the ideal *X*-directional first-order phase outputs after the shift without the influence of the additional positioning errors. The remaining terms correspond to the influence of the tilt, tip and piston errors. The phase output ${\tilde{I}}_{X\pm 1\_\mathrm{shifted}\_\mathrm{flat}}(x,y)$ after removing the least squares plane can be expressed as follows:

(A4) $${\tilde{I}}_{X\pm 1\_\mathrm{shifted}\_\mathrm{flat}}(x,y)=I_{X\pm 1\_\mathrm{shifted}}(x,y)-S_{h,X\pm 1\_\mathrm{shifted}}(x,y)$$

where *S_h,X_*_±1_shifted_(*x*, *y*) is a residual surface plane fitted to *I_X_*_±1_shifted_(*x*,*y*), after removing the least square plane *S_r,X_*_±1_shifted_(*x*, *y*) that can be expressed by the following equation:

(A5) $$S_{r,X\pm 1\_\mathrm{shifted}}(x,y)=a_{r,X\pm 1}x+b_{r,X\pm 1}y+c_{r,X\pm 1}=a_{X\pm 1}x+b_{X\pm 1}y+c_{X\pm 1}+S_{h,X\pm 1\_\mathrm{shifted}}(x,y)$$

where *a_r,X_*_±1_, *b_r,__X_*_±1_, and *c_r,__X_*_±1_ are the corresponding polynomial coefficients when fitting a plane to the measured phase outputs in Equation (A3). In the same manner, a least square plane can be fitted to the phase output obtained before the shift, and can be subtracted from the phase output as follows:

(A6) $${\tilde{I}}_{X\pm 1\_\mathrm{flat}}(x,y)=I_{X\pm 1}(x,y)-S_{h,X\pm 1}(x,y)$$

where *S_h,X_*_±1_(*x, y*) is the residual plane after removing the reference plane *S_r,X_*_±1_(*x*, *y*) from the phase output before the shift. It should be noted that the VLS grating moves over a small distance, comparable to one pixel of the imaging sensor of the Fizeau interferometer. Since the change in the overall profile of the diffracted wavefront after the small shift is not significant in the field of view, the following relationship can be found between the two residual planes:

(A7) $$S_{h,X\pm 1}(x,y)≅S_{h,X\pm 1\_\mathrm{shifted}}(x,y)$$

Consequently, the following relationship holds when applying the proposed reference plane technique:

(A8) $$\begin{array}{ll} {\tilde{I}}_{X\pm 1\_\mathrm{shifted}}(x,y)-{\tilde{I}}_{X\pm 1}(x,y) & =[I_{X\pm 1\_\mathrm{shifted}}(x,y)-I_{X\pm 1}(x,y)]+[S_{h,X\pm 1}(x,y)-S_{h,X\pm 1\_\mathrm{shifted}}(x,y)]\\ & ≅I_{X\pm 1\_\mathrm{shifted}}(x,y)-I_{X\pm 1}(x,y) \end{array}$$

Equation (A8) indicates that the additional tilt/tip/piston errors generated in the shifting process can be reduced by applying the proposed method. In the same manner, the analysis of the *Y*-directional first-order phase outputs can be carried out.
